# Regulating N Species in N‐Doped Carbon Electro‐Catalysts for High‐Efficiency Synthesis of Hydrogen Peroxide in Simulated Seawater

**DOI:** 10.1002/advs.202302446

**Published:** 2023-09-28

**Authors:** Nan Wang, Shaobo Ma, Ruiyong Zhang, Lifei Wang, Yanan Wang, Lihui Yang, Jianhua Li, Fang Guan, Jizhou Duan, Baorong Hou

**Affiliations:** ^1^ CAS Key Laboratory of Marine Environmental Corrosion and Bio‐Fouling Institute of Oceanology Chinese Academy of Sciences 7 Nanhai Road Qingdao 266071 China; ^2^ Science Center for Material Creation and Energy Conversion Institute of Frontier and Interdisciplinary Science Shandong University Qingdao 266237 China

**Keywords:** antibacterial, hydrogen peroxide, N‐doped carbon, oxygen reduction reaction, simulated seawater

## Abstract

Electrochemical oxygen reduction reaction (ORR) is an attractive and alternative route for the on‐site production of hydrogen peroxide (H_2_O_2_). The electrochemical synthesis of H_2_O_2_ in neutral electrolyte is in early studying stage and promising in ocean‐energy application. Herein, N‐doped carbon materials (N‐C_x_) with different N types are prepared through the pyrolysis of zeolitic imidazolate frameworks. The N‐C_x_ catalysts, especially N‐C_800_, exhibit an attracting 2e^−^ ORR catalytic activity, corresponding to a high H_2_O_2_ selectivity (≈95%) and preferable stability in 0.5 m NaCl solution. Additionally, the N‐C_800_ possesses an attractive H_2_O_2_ production amount up to 631.2 mmol g^−1^ h^−1^ and high Faraday efficiency (79.8%) in H‐type cell. The remarkable 2e^−^ ORR electrocatalytic performance of N‐C_x_ catalysts is associated with the N species and N content in the materials. Density functional theory calculations suggest carbon atoms adjacent to graphitic N are the main catalytic sites and exhibit a smaller activation energy, which are more responsible than those in pyridinic N and pyrrolic N doped carbon materials. Furthermore, the N‐C_800_ catalyst demonstrates an effective antibacterial performance for marine bacteria in simulated seawater. This work provides a new insight for electro‐generation of H_2_O_2_ in neutral electrolyte and triggers a great promise in ocean‐energy application.

## Graphical abstract

The synthesized graphite nitrogen doped carbon material delivers an attracting 2e^−^ ORR catalytic activity in 0.5 m NaCl simulated seawater solution, corresponding to a high H_2_O_2_ selectivity (≈95%), preferable stability and H_2_O_2_ production amount up to 631.2 mmol g^−1^ h^−1^. Additionally, the prepared catalyst demonstrates an effective antibacterial performance for marine bacteria in simulated seawater.

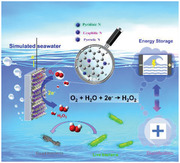

## 1 Introduction

H_2_O_2_ as a versatile clean oxidative chemical, is widely used for the degradation of organic dyes^[^ ^1^ ^]^ and organic drugs,^[^ ^2^ ^]^ water treatment,^[^ ^3^ ^]^ bacteria killing/ disinfection,^[^ ^4^ ^]^ and energy storage,^[^ ^5^ ^]^ due to the only byproduct of water without hazardous residues. At present, H_2_O_2_ is commonly synthesized through an energy‐intensive anthraquinone oxidation‐reduction in industrial scale.^[^ ^6^ ^]^ However, this method requires complex infrastructure and produces a substantial volume of organic byproduct wastes. Therefore, it is greatly important to develop highly efficient techniques for H_2_O_2_ synthesis. The direct synthesis of H_2_O_2_ through H_2_ and O_2_ is a straightforward and atom‐economic method.^[^ ^7^ ^]^ Nevertheless, this method causes the potential explosion hazard of H_2_/O_2_ mixtures.^[^ ^8^ ^]^ Electrocatalytic ORR is an attractive and alternative route for on‐site production of H_2_O_2_, which processes the advantages of low‐cost and convenience for in‐situ H_2_O_2_ generation at various application situations.

Substantial efforts in recent years have shown that electrocatalytic ORR is desirable and applicable for selective generation of H_2_O_2_.^[^ ^9^ ^]^ However, the ORR always occurs according to four‐electron process to generate H_2_O, which is a strong completive reaction to generate H_2_O_2_. Developing highly efficient electro‐catalyst is crucial to refining the H_2_O_2_ generation via 2e^−^ ORR. The previous reports indicate that noble metal‐based catalysts show high selectivity toward H_2_O_2_ under strongly acidic conditions, and transition metal‐based catalysts exhibit high 2e^−^ catalytic selectivity under strongly acidic or basic solutions.^[^ ^10^ ^]^ However, those noble/ transition metal catalysts always present low catalytic selectivity in neutral electrolytes.^[^ ^11^, ^12^ ^]^ Non‐metallic carbon‐based materials deliver good H_2_O_2_ selectivity in neutral electrolytes. Meanwhile, they process the advantages of high conductivity/stability, high mass transfer porosity, and low cost.^[^ ^13^ ^]^ Therefore, it is a promising 2e^−^ ORR catalyst, especially for large‐scale application. Heteroatom‐doping, especially N‐doping, is a useful strategy to improve the 2e^−^ ORR catalytic performance of non‐metallic carbon‐based catalysts by refining the catalytic active sites.^[^ ^14^, ^15^ ^]^ Some previous reports have shown that nitrogen‐doped carbons are in favor of 2e^−^ ORR catalytic activity in neutral phosphate buffer solutions.^[^ ^16^, ^17^ ^]^ However, the 2e^−^ ORR catalytic active sites of nitrogen‐doped carbons, such as pyridinic N, pyrrolic N and graphite N, are still controversial and remain a matter of active debates.^[^ ^18^, ^19^ ^]^ Accurately refining the N‐species in N‐doped carbon materials and clarifying the catalytic mechanism are crucial to guide and optimize the synthesis of efficient 2e^−^ ORR catalysts.

Noteworthily, most researchers focus on the electrochemical generation of H_2_O_2_ in strongly acidic or alkaline electrolyte, while the studies in neutral electrolyte are paid little attention.^[^ ^20^ ^]^ H_2_O_2_ synthesis in neutral solution is actually very useful and flexible for practical application of H_2_O_2_, as it can avoid the influence of pH.^[^ ^21^ ^]^ Seawater as an earth‐abundant resource stores pretty rich energy, and is investigated as an attractive electrolyte in energy‐field recently.^[^ ^22^ ^]^ It is also a promising neutral electrolyte for in site electrocatalytic production of H_2_O_2_. Meanwhile, the as‐synthesized H_2_O_2_ displays great application potential in marine biofouling field for sterilization. However, it is still in the early stages that the research on the use of seawater as electrolyte in energy system. Generally, 0.5 m NaCl is used as simulated seawater in the lab‐scale research.^[^ ^23^ ^]^ Whereas, the 2e^−^ ORR catalytic performance and catalytic mechanism of catalysts in simulated seawater electrolyte are still unsatisfactory and ambiguous. Thus, it is urgent to explore suitable catalysts and recognize the 2e^−^ ORR catalytic mechanism in neutral simulated seawater electrolyte.

Herein, N‐doped carbon materials are prepared using the facile pyrolysis method through calcinating Zeolitic imidazolate frameworks (ZIF‐8). The N species in the nitrogen‐doped carbon materials can be well refined by ranging the calcination temperature from 600 to 1000 °C. The structure, the species and content of N doping of resulting product are characterized by different analysis method. N‐doped carbon materials catalysts are investigated the effect of the different N doping on 2e^−^ ORR activity, selectivity, and stability in the neutral electrolyte. The result shows that N‐doped carbon exhibit remarkable electrochemical performance, which is attributed to the species and content of nitrogen‐doped carbon structure. In addition, density functional theory (DFT) was calculated, and the results showed that the species of nitrogen‐doped carbon structure are active sites, and conducive to 2e^−^ ORR. The amount of H_2_O_2_ generation were measured, and used to study the effect on the marine typical bacteria (*Pseudomonas aeruginosa*) in the simulated seawater. This study will provide an efficient alternative for water disinfection and other important application in the future.

## 2 Results and Discussion

### 2.1 Characterization

**Figure 1:**
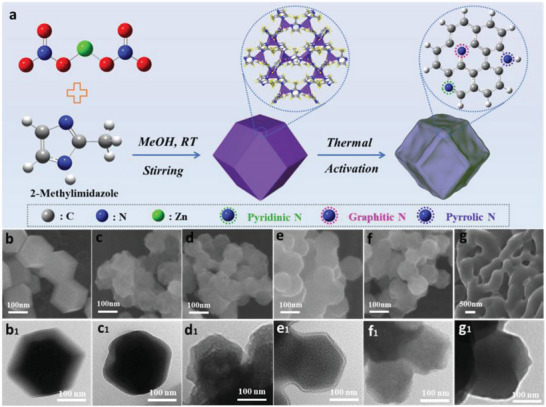
a) Synthesis schematic of nitrogen‐doped carbon materials, SEM and TEM images: b) and b_1_) ZIF‐8, c–g) and c_1_–g_1_) N doped carbon materials from annealing ZIF‐8 under 600, 700, 800, 900, and 1000 °C.

ZIF‐8 as a class of metal‐organic framework contains 34% of N‐containing 2‐methylimidazole linker,^[^ ^24^ ^]^ which is a good template and reactive precursor for nitrogen‐doped carbon materials. As shown in **Figure** **1**a, the N‐doped carbon materials were synthesized by a two‐step strategy, including the fabrication of high N content ZIF‐8 precursor and a further thermal carbonization and acid treatment activation process. The N‐C materials with different nitrogen‐doped types were obtained by regulating the annealing temperature of ZIF‐8 precursor. As shown in Figure 1b, the synthesized ZIF‐8 displays a regular and rhomb dodecahedral morphology. It is also observed in the corresponding TEM image (Figure 1b_1_). The N‐C_x_ materials exhibit the different degrees of shrinkage with the increasing pyrolysis temperature in Figure 1c–g. Generally, the zinc atoms in the ZIF‐8 frameworks present the different degrees of volatilization at various pyrolysis temperatures,^[^ ^25^ ^]^ which can cause the collapse of organic molecular skeleton. However, they still remain their basic polyhedron structure below the pyrolysis temperature of 900 °C, which can be observed by the corresponding TEM images (Figure 1c_1_–g_1_). Interestingly, the new structure was formed by the single rhomb dodecahedral accumulation, overlay and bond at the high temperature (1000 °C). It indicates that the framework structure is deformed and reorganized after the full volatilization of zinc atoms at this temperature. The above results demonstrate that the morphology/ micro‐structure of the N‐C_x_ materials is closely associated with the polymerization temperature and the volatilization of zinc atoms and is not affected by acid treatment.

**Figure 2:**
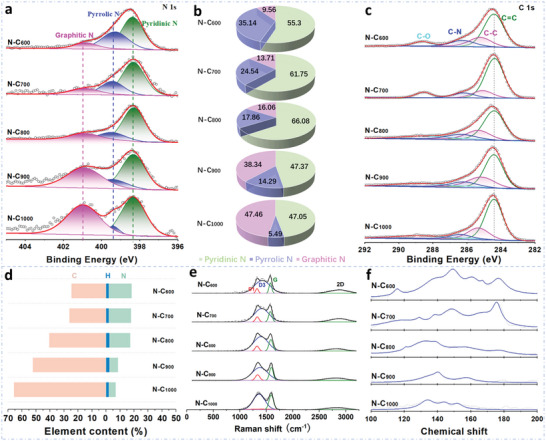
The structure and content characterization of the nitrogen‐doped carbon materials. a) High‐resolution XPS spectra N 1s. b) The relative amount of the different nitrogen species from the N 1s. c) High‐resolution XPS spectra C 1s. d) The content of elements. e) Normalized and baseline corrected Raman spectra. f) The NMR of the N‐C_x_ materials.

The high‐resolution XPS spectra of N‐doped carbon materials were further conducted to identify the binding mode of nitrogen and carbon atoms in the materials. The XPS survey spectra deliver typical C 1s, N 1s, O 1s, and Zn 2p signals (Figure S1, Supporting Information). As shown in **Figure** **2**a, the N 1s XPS spectrum can be deconvoluted into three peaks at about 398.4, 399.6, and 400.9 eV, which are assigned to pyridinic N, pyrrolic N and graphitic N, respectively.^[^ ^26^ ^]^ The accurate amounts of different N‐species are summarized in Figure 2b. The relative amount of pyrrolic N decreases whereas the relative amount of graphitic N increases with the increasing of annealing temperature, which is attributed to the stability of graphitic N being superior to pyrrolic N at the high annealing temperature.^[^ ^26^ ^]^ With the difference, the relative amount of pyridinic N increases first and then decreases with the increasing annealing temperature. It suggests that pyridinic N is easier to form than graphitic N at the low annealing temperature (<800 °C), while graphitic N is more stable at the higher annealing temperature (>800 °C).^[^ ^27^ ^]^ The high‐resolution C 1s XPS spectra show the presence of C═C (284.6 eV), C─C (285.4 eV), C─N (286.4 eV), and O─C═O (289.1 eV) functional groups (Figure 2c).^[^ ^28^ ^]^ The O1s spectra of the samples can be assigned to C─O (532 eV) and C═O (533 eV) bond, respectively (Figure S2, Supporting Information). With the increasing of pyrolysis temperature, the ratio of C─N and C─O bond content decrease and that of C─C bond raise, due to the destruction of the ZIF‐8 framework at high temperature. Figure 2d presents the change of elemental contents (N, C, and H) in different N‐doped carbon materials with the increasing annealing temperature. The accurate contents of N, C, and H are shown in Table S1 (Supporting Information). It is obvious that the H content remained almost constant (near 2 wt.%) in all different N‐doped carbon materials. However, the C content significantly increases due to the evaporating of the zinc with the temperature increasing. The N content just slightly decreases from 16.27% of N‐C_600_ to 14.81% of N‐C_800_. As the annealing temperature continues to increase, the N content presents an obvious decrease. Because zinc is stable in the formation of “Zn‐N”, the volatilization of Zn atoms at high temperature also takes away part of N.^[^ ^29^ ^]^ The above result is consistent with the results of XPS spectra N 1s (Figure 2a) and C1s (Figure 2c). Additionally, the Zn contents in all five samples are near 0.5–1.3 at.% (Table S2, Supporting Information) according to the quantitative XPS analysis results. The ICP‐OES results indicate that Zn content in all the five samples deliver comparable of near 2.1–2.8 wt.% (Table S3, Supporting Information). These results show that Zn content in all the five samples is low and comparable.

To further explore the structure of nitrogen‐doped carbon catalysts, the Raman spectra were measured using a 532 nm solid‐state laser as an excitation source. The D‐band is associated with the defects and G bands represent to C═C stretching vibrations of carbon layers related to the sp^2^ hybridizations.^[^ ^30^ ^]^ As shown in Figure 2e, the D peaks of the samples consist of two defect peaks at 1325 cm^−1^ (D1) and 1425 cm^−1^ (D3), which are attributed to defective edge carbon and amorphous sp^2^ carbon defects.^[^ ^31^ ^]^ The 2D at around 2880 cm^−1^ is also defined, which attributes to the presence of structural defects. With the calcined temperature increasing, there is a slight change between D1 and D3 signals due to the conversion of defective edge carbon and amorphous sp^2^ carbon defects.^[^ ^32^ ^]^ Furthermore, the I_D1+D3_/I_G_ ratio (Table S4, Supporting Information) of the N‐C_x_ catalysts display a decreasing tendency, which is associated with the increased graphitic degree of the N‐C_x_ catalysts at high annealing temperature.

The variation of N‐C_x_ catalysts in Raman spectra are highly consistent with the results in elemental contents (Figure 2d), XPS (Figure 2c) and TEM (Figure 1) measurements. In addition, the electrical conductivity of N‐C_x_ significantly increases with the improved thermal‐pyrolysis temperature (Table S5, Supporting Information). The enhanced electrical conductivity is also associated with their improved graphitization degree, which is well consistent with the XPS (Figure 2a) and Raman results (Figure 2e).

Solid‐state cross‐polarization/ magic angle spinning nuclear magnetic resonance (CP/MAS NMR) ^13^C spectra were then recorded to determine the chemical structure of the prepared samples. As displayed in Figure 2f, all the samples calcined at different temperature deliver a broad peak with the chemical shift of 100–150 ppm, which can be assigned to the sp^2^‐hybridized carbon.^[^ ^33^ ^]^ Especially, the spectra featured strong peaks at ≈ 130 and 155 ppm, which correspond to the aromatic carbon and graphite C═N groups.^[^ ^34^ ^]^ For the samples calcined below 800 °C, two distinct C signals are observed at around 125 and 170 ppm in the ^13^C NMR spectra, which are attributed to C≡N and carbonyl groups (C═O/COOH). Meanwhile, the intensity of the peaks at 125 and 170 ppm decreases as the carbonation temperature increases, implying a higher carbonization temperature can cause further aromatization and N content reduction. The above result is well consistent with the XPS result (Figure 2a) and element content analysis (Figure 2c). The BET measurements were conducted to investigate the specific surface area and pore size distribution of the materials (Figure S3, Supporting Information). The N‐C_x_ samples mainly display mesoporous structure and the specific surface area of the N‐C_600_, N‐C_700_, N‐C_800_, N‐C_900_ and N‐C_1000_ is 43, 56, 434, 1088 and 438.2 m^2^ g^−1^, respectively.

### 2.2 Electrochemical Property

**Figure 3:**
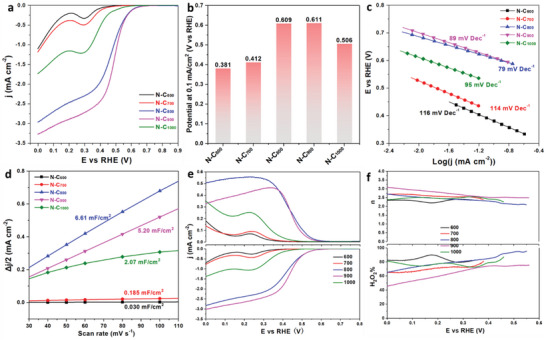
ORR performance of N‐doped carbon materials. a) ORR polarization curves in O_2_‐saturated 0.5 m NaCl (rotation rate: 1600 rpm, sweep rate: 10 mV s^−1^), b) Bar plots of *E*_onset_, c) Tafel plots derived from panel (a), and d) Current density as a function of scan rate. e) linear sweep voltammetry performed by a RRDE technique where the ring current is collected on the Pt ring at a constant potential of 1.5 V_RHE_ and f) calculated n and H_2_O_2_ selectivity (%), as a function of electrode potential.

To reveal the electrochemical behavior of N‐C_x_ catalysts, CV curves were measured in O_2_ or N_2_‐saturated 0.5 m NaCl solution. All the potentials in this work were referenced to the reversible hydrogen electrode (RHE). The CV curves indicate an obvious oxygen reduction peak in O_2_‐saturated electrolyte compared with in N_2_‐saturated electrolyte (Figure S4, Supporting Information). The ORR catalytic activity of N‐C_x_ catalysts were measured using the RDE in an O_2_‐saturated 0.5 m NaCl solution (pH = 6.88). **Figure** **3**a shows the ORR polarization curves of the N‐doped carbon materials with the annealing temperature from 600 to 1000 °C. Similar with the results of CV, the LSV curves exhibit an obvious oxygen reduction peak in the O_2_‐saturated 0.5 m NaCl solution. Apparently, the current densities of N‐C_600_ and N‐C_700_ are significantly lower than those of other catalysts, which maybe is related to their low content of graphitic N and the poor conductivity. Some reports indicate that graphitic N is responsible for the ORR activity of N‐doped carbons.^[^ ^35^ ^]^ The inferior LSV curves and catalytic performance of the N‐C_600_, N‐C_700_ and N‐C_1000_ are associated with their poor catalytic sites, electrical conductivity, and specific surface area.^[^ ^36^, ^37^ ^]^ Figure 3b displays the values of the onset potential for various catalysts, which are selected at a current density of 0.1 mA cm^−2^ as the previous reports.^[^ ^38^ ^]^ Notably, the N‐C_800_ and N‐C_900_ exhibit an outstanding and almost identical onset potential (*E*_onset_) of about 0.61 V (vs RHE). Their values are much more positive than N‐C_600_ (0.381 V) and N‐C_700_ (0.412 V), even much more positive than N‐C_1000_ (0.506 V). The ORR catalytic kinetics of the catalysts are further estimated by the Tafel plots (Figure 3c). The N‐C_800_ displays a smaller Tafel slope (79 mV decade^−1^) than N‐C_900_ (89 mV decade^−1^), N‐C_1000_ (95 mV decade^−1^), N‐C_700_ (114 mV decade^−1^) and N‐C_600_ (116 mV decade^−1^), indicating a superior catalytic kinetic of the N‐C_800_, which is consistent with the results of onset potentials. Furthermore, to establish the intrinsic activity of the N‐C_x_ catalysts, electrochemical double‐layer capacitance (C_dl_) was investigated by CV at different sweep rates (Figure S5, Supporting Information). The C_dl_ of N‐C_x_ catalysts were calculated and shown in Figure 3d. The results demonstrate N‐C_800_ possesses a significantly larger ECSA than other N‐C_x_ catalysts, which greatly contributes to its ORR catalytic performance. The above results indicate that N‐C_800_ delivers the best 2e^−^ ORR catalytic activity among various N‐C_x_ catalysts.

The catalytic selectivity of N‐C_x_ catalysts is the key electrocatalytic performance for 2e^−^ ORR. It is further investigated using RRDE in an O_2_‐saturated 0.5 m NaCl solution. Figure 3e shows the RRDE curves of the N‐C_x_ catalysts, in which the ORR current and the simultaneous H_2_O_2_ detection current are obtained on the disk electrode and Pt ring electrode, respectively. The N‐C_800_ exhibits the highest ring current among all the N‐C_x_ catalysts. It is attributed to its abundant N content and graphite‐N type in N‐C_800_ catalyst. Meanwhile, the N‐C_800_ achieves almost the highest ORR disk current (comparable with that of N‐C_900_) among those N‐C_x_ catalysts. Therefore, the N‐C_800_ presents a superior performance, not only in catalytic activity but also in catalytic selectivity for H_2_O_2_ production. Figure 3f shows the catalytic selectivity for H_2_O_2_ production with the number of transferred electrons (n) and H_2_O_2_ selectivity (H_2_O_2_%). For all the N‐C_x_ materials, both H_2_O_2_ selectivity and n variation tendency depend on the applied potential.^[^ ^10^ ^]^ The corresponding n value is given near two, which is consistent with the H_2_O_2_ selectivity, suggesting N‐C_x_ catalysts mainly follow 2e^−^ oxygen reduction pathway. The N‐C_800_ exhibits a high H_2_O_2_ selectivity at the whole potential range. Especially, the H_2_O_2_ selectivity of N‐C_800_ is above 90% at a high potential range (>0.446 V), which is better than other catalysts. The N‐C_800_ also exhibits a comparable H_2_O_2_ selectivity at the low potential (<0.446 V). Additionally, the N‐C_800_ sample was immersed into aqua regia solution (HNO_3_: HCl = 1:3) for 12 h to fully remove the Zn species, which was labeled as N‐C_800_ (0 wt.% Zn). Compared to N‐C_800_ (0 wt.% Zn), the N‐C_800_ delivers an almost same onset potential, a slightly higher disk current density and lower 2e^−^ ORR selectivity (Figures S6 and S7, Supporting Information). Both the N‐C_800_ and N‐C_800_ (0 wt.% Zn) deliver an attracting and similar 2e^−^ ORR catalytic performance, implying the catalytic effect of Zn concentration in this N‐C_x_ catalyst is weak. These results confirm that the N types of the N‐C_x_ are crucial to the 2e^−^ ORR selectivity.

**Figure 4:**
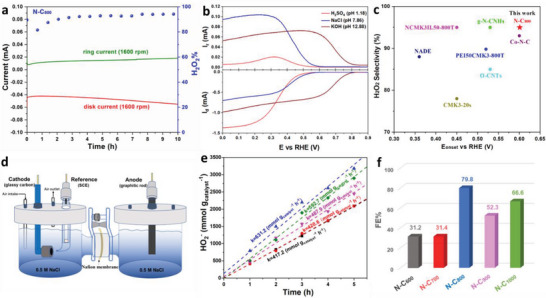
a) Stability tests of N‐C_800_ at 0.51 V versus RHE, b) LSV of N‐C_800_ recorded in 0.1 m H_2_SO_4_, 0.5 m NaCl and 0.1 m KOH at 1600 rpm, with the ring electrode at a constant potential of 1.5 V_RHE_, c) Comparison of the onset potential and H_2_O_2_ selectivity in the neutral solutions, d) H‐Cell electrolyzer for H_2_O_2_ production in 0.5 m NaCl solutions, e) H_2_O_2_ production amount normalized to catalyst loading amount over the reactive time at applied potentials and f) The Faraday efficiency of H_2_O_2_ for N‐C_x_ catalysts under the flow of O_2_.

The stabilities of N‐C_x_ materials were further studied in O_2_‐saturated 0.5 m NaCl under their optimal applied potential according to H_2_O_2_ selectivity. As shown in **Figure** **4**a, the current of the disk/ ring electrode and H_2_O_2_ selectivity of the N‐C_800_ remain pretty stable for 10 h without an obvious decay. It indicates that the N‐C_800_ catalyst keeps a high structural and 2e^−^ ORR catalytic stability during the electrochemical process. Meanwhile, the other catalysts also deliver comparable stabilities at their optimal applied potentials (Figure S8, Supporting Information). The results indicate that N‐C_800_ catalyst presents good catalytic activity, selectivity and stability at the same time. In addition, the electrocatalytic activity and H_2_O_2_ selectivity of N‐C_x_ were tested in 0.1 m H_2_SO_4_ and 0.1 m KOH electrolyte solution with different pH values. Figure 4b shows the 2e^−^ ORR catalytic performance of N‐C_800_ in 0.1 m H_2_SO_4_, 0.5 m NaCl and 0.1 m KOH electrolyte solution, separately. The results show that N‐C_800_ presents high disk currents in above three electrolyte solutions, illustrating a good catalytic activity. Meanwhile, the ring current tested in 0.5 m NaCl is the highest, indicating the best H_2_O_2_ selectivity (Figure S9, Supporting Information). The other types of N‐C_x_ catalyst also present the best H_2_O_2_ selectivity in 0.5 m NaCl solution (Figures S10–S13, Supporting Information). In a neutral electrolyte, the 2e^−^ ORR catalytic performance of prepared catalyst in this work is compared with those in previous reports (Figure 4c; Table S6, Supporting Information).^[^ ^9^, ^13^, ^16^, ^22^, ^23^, ^26^, ^39^ ^]^ It is clear that the N‐C_800_ catalyst exhibits a better/ attracting onset potential and H_2_O_2_ selectivity, which are even higher than those of some metal‐based catalysts (Co─N─C).

The H_2_O_2_ yield is a crucial parameter to evaluate the catalytic performance of the materials especially in practical applications. Herein, an H‐Cell electrolyzer is used for producing H_2_O_2_ according to ORR process in 0.5 m NaCl solutions (Figure 4d). The generation amount of H_2_O_2_ can be detected through a photometric method.^[^ ^13^ ^]^ Based on the standard curve of H_2_O_2_ concentration measurement by cerium method (Figure S14, Supporting Information), Figure 4e displays the accumulated amounts of H_2_O_2_ for various catalysts in O_2_‐saturated 0.5 m NaCl, which are normalized by catalyst loading amount over the reaction time. Remarkably, for the H_2_O_2_ production of ≈410–630 mmol g^−1^ h^−1^, the catalytic rate of various catalysts delivers a list of N‐C_800_ > N‐C_1000_ > N‐C_900_ > N‐C_700_ > N‐C_600_, which is better than that of the reported nitrogen‐doped carbon catalyst.^[^ ^39^ ^]^ The results show that N‐C_800_ possesses the highest H_2_O_2_ production amount up to 631.2 mmol g^−1^ h^−1^. The H_2_O_2_ production amount of N‐C_900_ is lower than that of N‐C_1000_, which is attributed to the consecutive decomposition reaction of H_2_O_2_. The peroxide reduction reaction (PRR) result displays the N‐C_900_ catalyst a high cathodic current, indicating a H_2_O_2_ reduction tendency (Figure S15, Supporting Information). In addition, the Faraday efficiency of H_2_O_2_ is another important parameter for evaluating the performance of the catalyst. It was calculated according to the real amount of produced H_2_O_2_ and H_2_O_2_ selectivity of the catalysts in their investigated applied potential (Figure 4f). The result indicates that N‐C_x_ catalysts exhibit Faraday efficiency ranging from 31.2% to 79.8% H_2_O_2_. Especially, the N‐C_800_ catalyst stands the top level Faraday efficiency (79.8%) for H_2_O_2_ production.

### 2.3 Theoretical Calculations

**Figure 5:**
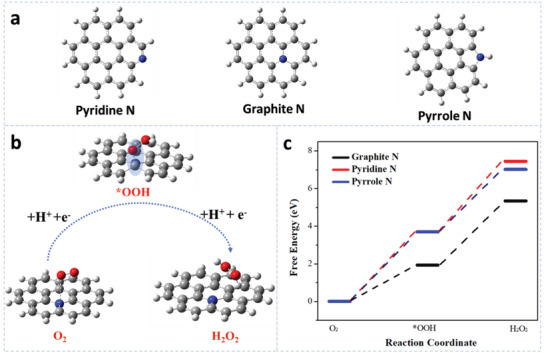
a) Schematic illustration of the three nitrogen doping configurations, b) The schematic of 2e^−^ ORR catalytic active sites from carbon adjacent to graphitic N and c) Free energy diagrams of 2e^−^ ORR on the different typical nitrogen doped carbon materials.

To further confirm the catalytic active sites of various N species in N‐C_x_ catalysts, the DFT calculations were carried out to evaluate the free energies of the adsorbed intermediate of the catalytic reaction. First, the geometrical structure of N‐graphene (pyridinic, pyrrolic or graphitic N) were optimized using vibration analysis in **Figure** **5**a. The optimized geometry structure is nearly perfect, indicating that these optimized structures are stable. The 2‐electron ORR mechanism is generally considered to proceed through the following steps (1 and 2):^[^ ^40^ ^]^

(1) $$*\;+O_{2}\;+H^{+}\;+{\;e}^{-}\to *\mathrm{OOH}$$

(2) $$*\mathrm{OOH}+H^{+}\;+e^{-}\to H_{2}O_{2}+*$$

where asterisks (*) indicate unoccupied active sites. *OOH denotes the important and single intermediate for the reaction. Among the adsorption energies between catalysts and *OOH intermediate determines the reaction product, which is recognized as the rate‐determining step.^[^ ^41^ ^]^ The DFT calculations work well in describing adsorption energies of intermediates on the different N‐graphene surface. As shown in Figure 5b, the geometrical structure of carbon adjacent to graphitic N obviously distorted after adsorbing O_2_ reactant and *OOH intermediate. Thus, the carbon adjacent to graphitic N is the main catalytic active site to promote H_2_O_2_ generation. The schematic illustration of pyridinic N and pyrrolic N for 2e‐ ORR were also given in Figures S16 and S17 (Supporting Information). In addition, the computational hydrogen electrode model (CHE) was used in the catalytic process.^[^ ^42^ ^]^ Figure 5c shows the DFT calculated free diagram of the various N‐graphene (pyridinic‐N, pyrrolic‐N and graphitic‐N) for the 2e^−^ ORR process using the CHE model. The free energy of various N‐graphene to catalyze the H_2_O_2_ generation delivers the order of pyridinic‐N > pyrrolic‐N > graphitic‐N. According to the DFT calculation, the graphitic‐N doped graphene displays the best 2e^−^ ORR catalytic performance, which is well consistent with the experimental results.

### 2.4 Antibacterial Properties

**Figure 6:**
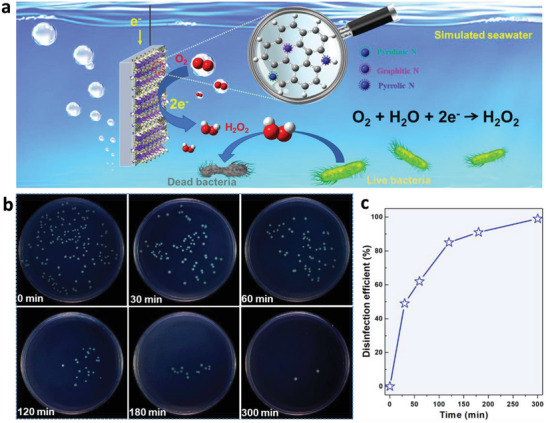
a) schematic of electrochemical synthesis of H_2_O_2_ for antibacterial. b) The disinfection efficiency as a function of treatment time. c) Photos of cultured plates with spread droplets taken from different time slots during the electrolysis. Dilution factor is labeled of each image.

Utilizing seawater as electrolyte can reduce the cost and broaden the on‐site production of H_2_O_2_ along the coast in energy‐related field. Meanwhile, H_2_O_2_ as an oxidizer has been used for bacteria killing.^[^ ^43^ ^]^ Exploring a kind of low‐cost catalyst for efficient H_2_O_2_ generation is very promising in the practical applications of the marine field, especially for antibacterial and antifouling of the offshore engineering facilities. In the current lab‐scale research, we conduct the disinfection properties of the N‐C material in 0.5 m NaCl solution to mimic the practical bacterial killing applications. The schematic of the electro‐chemical 2e^−^ ORR progress for bacteria killing in simulated seawater is shown in **Figure** **6**a. The sterilization effect of the prepared electro‐catalyst is further evaluated using a H‐cell electrolytic cell and *Pseudomonas aeruginosa* (*P. Aeruginosa*) marine bacteria. The obtained *P. Aeruginosa* concentration is ≈10^8^ c.f.u. mL^−1^ by plate counting and is operated with a suitable dilution magnification. The electrolyte with different concentrations of H_2_O_2_ is picked up at a series of time (0, 30, 60, 120, 180, and 300 min) during i–t measurement (0.51 V vs RHE). The picked electrolyte is further diluted before sterilization, and the images of agar plates with cultured bacteria colony after sterilization are displayed in Figure 6c. The number of colonies gradually decreases with the increasing of electro‐catalytic time (as more H_2_O_2_ is generated), and the number of colonies decreases to be almost negligible after 300 min. It is obvious that N‐C_800_ demonstrates a promising disinfection efficiency for *P. Aeruginosa* according to the calculated killing rate plotted (Figure 6b). These above results indicate that the synthesized N‐C electrocatalyst presents a good sterilization effect in the simulated seawater. This could provide an experimental basis for practical marine antibacterial and antifouling applications in the future.

## 3 Conclusion

In summary, the N‐C_x_ materials with different types of doped‐nitrogen (graphitic N, pyridinic N, and pyrrolic N) are synthesized by varying the pyrolysis temperatures of ZIF‐8. The N‐C_x_ catalysts exhibit a distinguishing 2e^−^ ORR performance in 0.5 m NaCl solution due to their different N‐doping content and types. Especially, N‐C_800_ exhibits the best catalytic activity with an onset potential of ≈0.6 V (vs RHE), catalytic selectivity with ≈95%, and high catalytic stability in 0.5 m NaCl solution. This correlates with the high ratio and content of graphitic N in N‐C_800_ catalysts. N‐C_800_ catalyst possesses a high H_2_O_2_ production amount up to 631.2 mmol g^−1^ h^−1^, and stands the top level Faraday efficiency (79.8%). The DFT calculation shows that the graphitic N displays higher 2e^−^ ORR catalytic activity than pyridinic N and pyrrolic N, and the carbon atoms adjacent to graphitic N act as the active sites to interact with reaction intermediates. The results strongly suggest that graphitic N is more favorable to the 2e^−^ ORR performance. In addition, the H_2_O_2_ generated by N‐C_800_ electro‐catalyst in simulated seawater displays a favorable sterilization effect, which is meaningful for in‐site bacteria killing in marine field. This work may provide a promising possibility for converting the marine resource to energy storage and microbial fouling protection.

## 4 Experimental Section

### Synthesis ZIF‐8

The ZIF‐8 framework was prepared by reacting 2‐Methylimidazole with Zn(NO_3_)_2_ at room temperature according to the previous report.^[^ ^44^ ^]^ Typically, 1.752 g of 2‐methylimidazole was dissolved in 200 mL methanol to form solution A. 7.93 g of Zn(NO_3_)_2_∙6H_2_O was dissolved in 200 mL of methanol to form solution B. Solution B was poured in solution A all at once. The mixture was kept under stirring for 24 h. The precipitate was separated by filtration and washed with methanol for three times. After drying under vacuum at 60 °C, the white powder precursors were obtained.

### Synthesis of N‐C Materials

The synthesized precursors were pyrolyzed at a series of temperatures (600, 700, 800, 900, and 1000 °C) for 2 h with the ramping rate of 2 °C min^−1^ under an argon atmosphere. The calcined samples were washed with moderate hydrochloric acid solution and labeled as N‐C_x_, where x was defined as the calcinated temperature.

### Characterization

The morphologies of ZIF‐8 and N‐C were characterized by scanning electron microscopy (SEM) and transmission electron microscopy (TEM) on HITCH microscope (Regulus and HT7700, Japan). The electronic structure and chemical bonding of materials were determined using the X‐ray photoelectron spectroscopy (XPS, K‐alpha 250Xi, England). The elemental contents of C, H, N, and S in the materials were conducted on an elemental analysis instrument (Elementar Unicube, EA 3000, Germany) and the Raman spectra were collected on Renishaw MZ20‐FC Raman microscope. Metal content were evaluted by the inductively coupled plasma‐optical emission spectrometer (ICP‐OES, iCAP RQ). Solid‐state nuclear magnetic resonance (NMR) of ^13^C was performed on the Bruker AVANCE III HD NMR 400MWB spectrometer, equipped with a 4.0 mm MASDVT BL4.0 X/Y/F‐H resonance probe head. Cross‐polarization/magic angle spinning (CP/MAS) sequence was performed to enhance the ^13^C NMR signal response. The synthesized powder samples were packed inside Zirconia MAS rotor with a diameter of 4.0 mm and a vespel cap. The ^13^C Hahn‐echo MAS spectra were acquired with a 2 s recycle delay, a ^13^C excitation (90^o^) pulse length of 2 s and 20 kHz MAS. The electrical conductivity of the materials was determined using two‐probe measurement method (ROOKO FT‐300L) at series of testing pressure (12, 15, or 20 MPa). The specific surface area of materials was measured with Brunauer–Emmett–Teller analyzer (BET, ASAP2460). The absorbance of the solutions was measured by UV–vis spectroscopy (HITCH 3900, Japan).

### Electrochemical Measurements

Electrochemical tests were performed using a CHI electrochemical workstation (CHI 760E) coupled with a rotating‐ring disc electrode (RRDE, Pine) in a three‐electrode cell. A graphite rod and a saturated calomel electrode (SCE) were used as the counter electrode and reference electrode, respectively. The reference electrode was calibrated to a reversible hydrogen electrode (RHE) before each measurement. All potentials measured against SCE were converted to the RHE scale using *E* (vs RHE) = *E* (vs SCE) + *E^ɵ^* (SCE)+ 0.059*pH, where *E^ɵ^* (SCE) value was the calibrated value, pH value of electrolytes was determined by the pH meter (lightning magnetism, PHSJ‐3F). A RRDE assembly (258051, Pine Instruments) consisting of a glass carbon rotation disk electrode (0.196 cm^2^) and a Pt ring (0.2475 cm^2^) was used, with the collection efficiency of 37%. The electrocatalysts inks with the mass concentration of 5 mg mL^−1^ catalysts were prepared by dispersing a certain amount of catalyst in an isopropanol solution with Nafion (5%). 6 µL of each catalyst ink was pipetted on a precleaned glass carbon disk electrode and dried at room temperature to yield a uniform thin‐film electrode.

The ORR catalytic activity was measured by linear sweep voltammetry (LSV) with a rotation rate of 1600 rpm in an O_2_‐saturated electrolyte solution (the solution resistance was compensated). Cyclic voltammetry (CV) with N_2_‐saturated 0.5 m NaCl solution were measured at the different scan rates to estimate the electrochemical active surface area (ECSA).^[^ ^45^ ^]^ ECSA was estimated according to electrochemical double‐layer capacitance (C_dl_), based on the positive proportional relationship between ECSA and C_dl_ (ECSA = C_dl_ /C_s_),^[^ ^46^ ^]^ where C_s_ is the specific capacitance of carbon materials, and its real value is unknown. C_dl_ was determined by conducting CV with a 3.5% NaCl electrolyte solution with a potential range of non‐faraday reaction at increasing scan rates of 5, 10, 20, 40, 50, 60, 80, 100, 150, 200, 250, 300 mV s^−1^. The value of C_dl_ was obtained from the slope of a derived plot of average current density versus the scan rate. The average current density is equal to (I_a_+I_c_)/2, where I_a_ and I_c_ are the anodic current and the cathodic current, respectively, which can be read from the CV curves.^[^ ^47^ ^]^

The catalytic selectivity was measured by an RRDE in an O_2_‐saturated electrolyte solution at a rotation rate of 1600 rpm. The Pt‐ring electrode was polarized at 1.2 V_RHE_ to further reduce the as‐formed H_2_O_2_ from the disk electrode. The electron transfer number and selectivity for the H_2_O_2_ yield were calculated from the disk current and ring current according to the corresponding formula.^[^ ^44^ ^]^ Among that, the current collection efficiency of the Pt‐ring was 0.37.

The stability of catalysts was determined by chronoamperometry with a rotation rate of 1600 rpm in an O_2_‐saturated 0.5 m NaCl solution at room temperature. Meanwhile, a potential of 1.2 V_RHE_ was used to the Pt‐ring electrode during the entire testing process. It is noted that the pH value remained almost unchanged before and after the continuous electrolysis.

H_2_O_2_ production in 0.5 m NaCl was carried out in the H‐cell electrolyzer. The cathode anode chambers were separated with Nafion 117 membrane. 6 µL of each catalyst ink was loaded on working electrode. A chronoamperometry measurement was performed to H_2_O_2_ production. The H_2_O_2_ concentration was quantified by cerium sulfate titration method.^[^ ^13^ ^]^ A yellow solution of Ce^4+^ would be reduced by H_2_O_2_ to colorless Ce^3+^ (2Ce^4+^ + H_2_O_2_ — 2Ce^3+^ + 3H^+^ + O_2_). Based on this mechanism, the concentration of Ce^4+^ before and after the reaction can be measured by UV–vis spectroscopy. The characteristic absorption peaks appear at wavelength of 316 nm. The 1 mm Ce(SO_4_)_2_ solution was prepared by previous reported.^[^ ^13^ ^]^ The calibration curve of H_2_O_2_ were determined by measuring the mixture of known concentration H_2_O_2_ and Ce(SO_4_)_2_ solution. Based on the linear relationship between the signal absorption value and know concentration H_2_O_2_, the H_2_O_2_ concentrations of the samples could be obtained.

### Computational Details

The density functional theory (DFT) was conducted using the Guassian09 program package to simulate the mechanistic process of O_2_ reduction reaction on various N‐graphene substrates. No geometric constraints were assumed in geometry optimization. The nonlocal correlation functional of Lee, Yang, and Parr^3^ (B3LYP) with the 6–31++G** basis set was used for C, N, H and O atom. The pristine graphene, with 7 hexagonal rings with delocalized π electron, was used as the baseline model. With the same configuration as pristine graphene, N‐graphene with three different N‐graphene models were constructed, including pyridinic N, pyrrolic N, and graphitic N. The hydrogen atoms terminate the carbon atoms at the edge of the graphene. The relative energies of the molecules presented in this study were zero‐point‐energy (ZPE) obtained from frequency calculations at the same level of optimization.

O_2_ reduction reaction was simulated, beginning with the adsorption of an O_2_ molecule onto an N‐graphene surface. In this step, we placed O‐O near the N‐graphene plane, then the structures of O─O adsorption on N‐graphene was optimized structures by the structural optimization calculations. The adsorption energy was derived from frequency analysis after the initial structure optimization. At the same process, the structure of OOH and HOOH adsorption on N‐graphene were optimized, respectively. Finally, the relative energy between the reactants and products was calculated. The other types of N‐graphene model were optimized and calculated at the same method.

### Antibacterial Tests

A typical marine bacterial *Pseudomonas aeruginosa* (*P.aeruginosa*) strain was provided by our laboratory. *P. Aeruginosa* was cultured in 2216E liquid medium at 37 °C for 24 h with 130 rpm shaking. 1 mL of the obtained green culture liquid was centrifugated at 4000 rpm to separate the bacterial biomass from the medium. Then, the bacterial cells were diluted and suspended in 0.1 m phosphate buffer saline (PBS) for plate counting. Others were added into 0.5 m NaCl. The electrochemical antibacterial measurements were run at room temperature in H‐type glass cell separated by Nafion 117 membranes. 50 mL of the prepared *P. Aeruginosa* (≈10^8^ c.f.u. mL^−1^) in 0.5 m NaCl were added into the cathodic chamber, and 50 mL of bacteria‐free 0.5 m NaCl were added into the anode chamber under sterile conditions. Glass carbon electrode (Ф = 5.0 mm) with 3 µg N‐C_800_ catalysts was served as the working electrode. A chronoamperometry curve measurement at the same voltage value (0.51 V_RHE_) was carried out to verify the antibacterial performance. The bacteria killing rates were measured using standard spreading plating techniques. 200 µL electrolyte with bacteria were taken at different time interval during the electrolysis process. Samples were then serially diluted and plated on 2216E agar plates in triplicate. Plates were incubated at 37 °C for 24 h or more to make the larger colony and easier to observe and count. The photos of cultured plates were taken with the fully automatic colony counter (icount 30, Hangzhou Xunshu Technology Co., Ltd).

## Conflict of Interest

The authors declare no conflict of interest.

## Supporting information

Supporting Information

## Data Availability

The data that support the findings of this study are available from the corresponding author upon reasonable request.
