# Supplementary material for: Regulating N Species in N‐Doped Carbon Electro‐Catalysts for High‐Efficiency Synthesis of Hydrogen Peroxide in Simulated Seawater
Source: Adv Sci (Weinh). 2023 Sep 28;10(31):2302446. doi: 10.1002/advs.202302446 (PMC10625060; doi:10.1002/advs.202302446)
Supplement: Supplementary file 1 — Supporting Information [file ADVS-10-2302446-s001.pdf]

ADVANCED SCIENCE
Open Access

Supporting Information

for *Adv. Sci.*, DOI 10.1002/advs.202302446

Regulating N Species in N-Doped Carbon Electro-Catalysts for High-Efficiency Synthesis of Hydrogen Peroxide in Simulated Seawater

Nan Wang, Shaobo Ma, Ruiyong Zhang, Lifei Wang, Yanan Wang, Lihui Yang, Jianhua Li, Fang Guan, Jizhou Duan* and Baorong Hou**

Supporting Information

Regulating N species in N-doped carbon electro-catalysts for high-efficiency synthesis of hydrogen peroxide in simulated seawater

Nan Wang[1], Shaobo Ma[2], Ruiyong Zhang[1], Lifei Wang[1], Yanan Wang[1], Lihui Yang[1], Jianhua Li[1], Fang Guan[1], Jizhou Duan[1]*, Baorong Hou[1]**

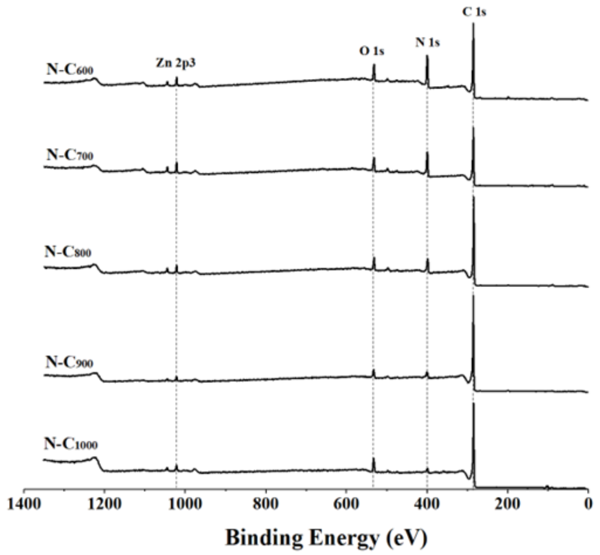

Figure s1 XPS survey spectra of N-C600, N-C700, N-C800, N-C900, and N-C1000.

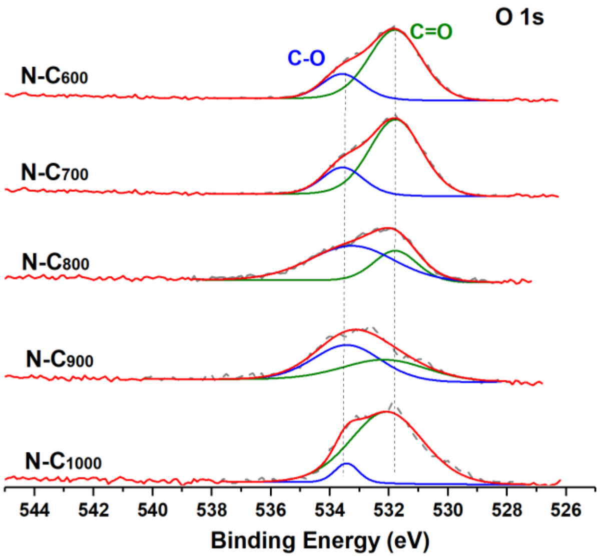

Table s1 The content of the elements in the different N-doped carbon materials.

samples	N-C_{600}	N-C_{700}	N-C_{800}	N-C_{900}	N-C_{1000}
N	16.27	15.86	14.81	6.04	4.89
C	24.73	26.17	40.58	52.38	65.83
H	2.09	2.112	2.5	2.591	2.12

Table s2 Zn content determined by XPS for N-C_x.

Catalysts	N-C_{600}	N-C_{700}	N-C_{800}	N-C_{900}	N-C_{1000}
Zn content in N-C_x	0.83%	1.29%	0.9%	0.54%	0.71%

Table s3 Zn content determined by ICP for N-C_x.

Catalysts	N-C_{600}	N-C_{700}	N-C_{800}	N-C_{900}	N-C_{1000}
Zn content in N-C_x	2.56%	2.79%	2.30%	2.07%	2.15%

Table s4 the integral intensity (area) percentage and their ratio of the different N-doped carbon materials

Samples	I_{D1}	I_{D3}	I_G	I_{D1+D3}/I_G
N-C_{600}	4.29759	65.72042	9.4622	7.39976
N-C_{700}	5.81674	63.50906	11.62392	5.964064
N-C_{800}	5.99079	67.17099	14.50265	5.044718
N-C_{900}	7.62488	63.10856	15.37074	4.601824
N-C_{1000}	3.98871	61.4833	22.13119	2.958359

Table s5 the electrical conductivity of the N-C_x.

Catalysts	Electrical conductivity (mS/mm)		
	12 MPa	15 MPa	20 MPa
N-C_{600}	$8.21*10^{-6}$	$1.746*10^{-5}$	$3.689*10^{-5}$
N-C_{700}	$5*10^{-5}$	$6*10^{-5}$	$7*10^{-5}$
N-C_{800}	0.15627	0.17014	0.18867
N-C_{900}	15.49	16.75	18.92
N-C_{1000}	310	350	400

Figure s2 High-resolution XPS spectra O 1s.

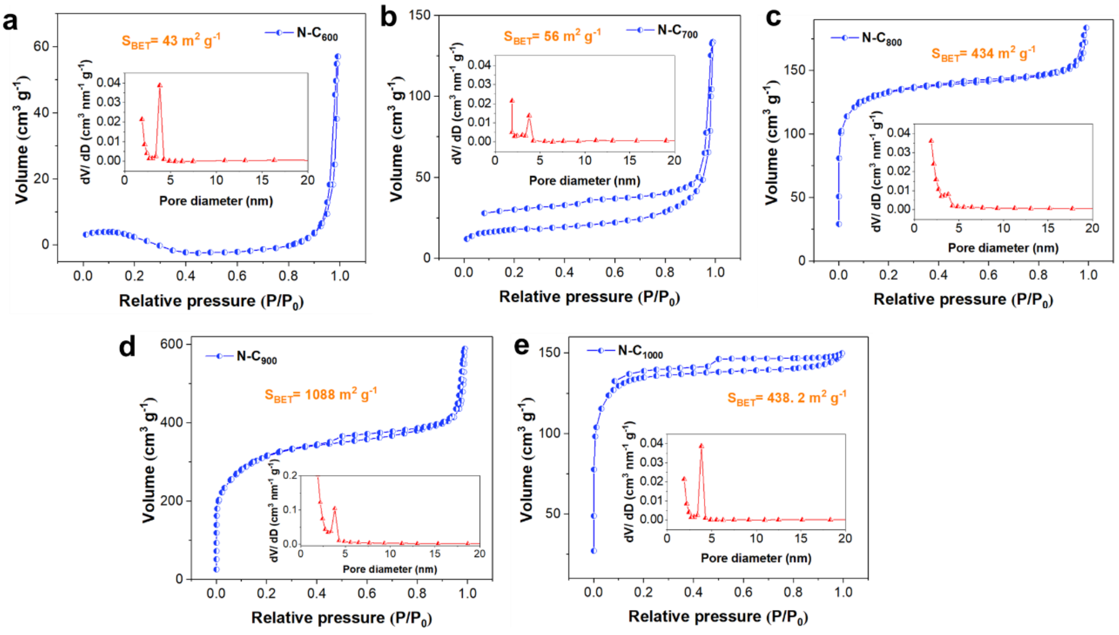

Figure s3 (a-e) BET analysis results of the N-C_x. The insets show their corresponding pore sizes.

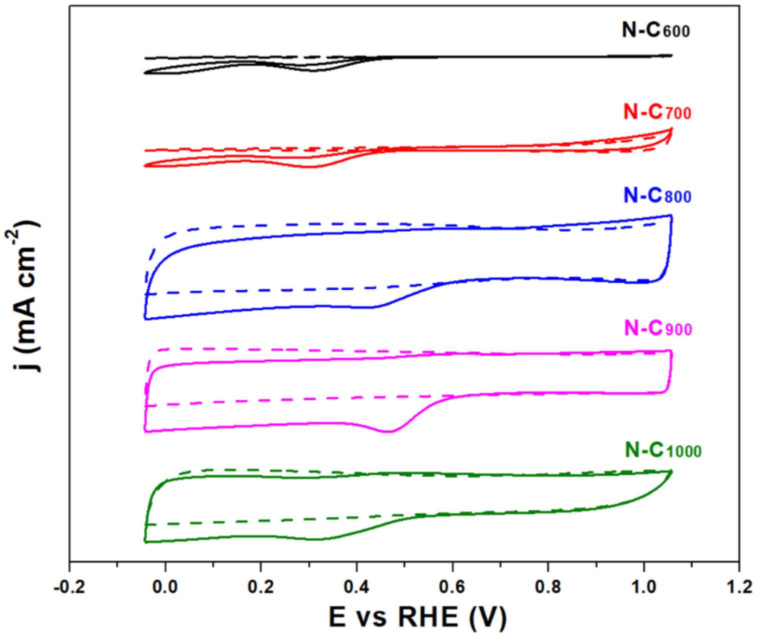

Figure s4 CV curves at 50 mV s^{-1} in 3.5% NaCl solution under saturated O_2 (solid line) and N_2 (dash line).

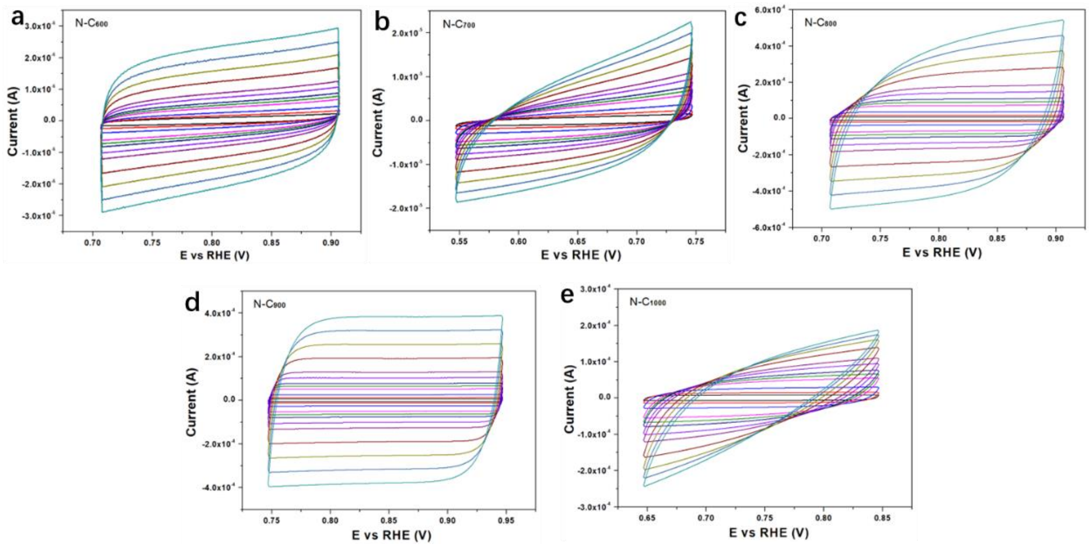

Figure s5 (a-e) CV curves at different sweep rates (5, 10, 20, 40, 50, 60, 80, 100, 150, 200, 250, 300 mV s^{-1}) for the N-C_{600}, N-C_{700}, N-C_{800}, N-C_{900}, and N-C_{1000}.

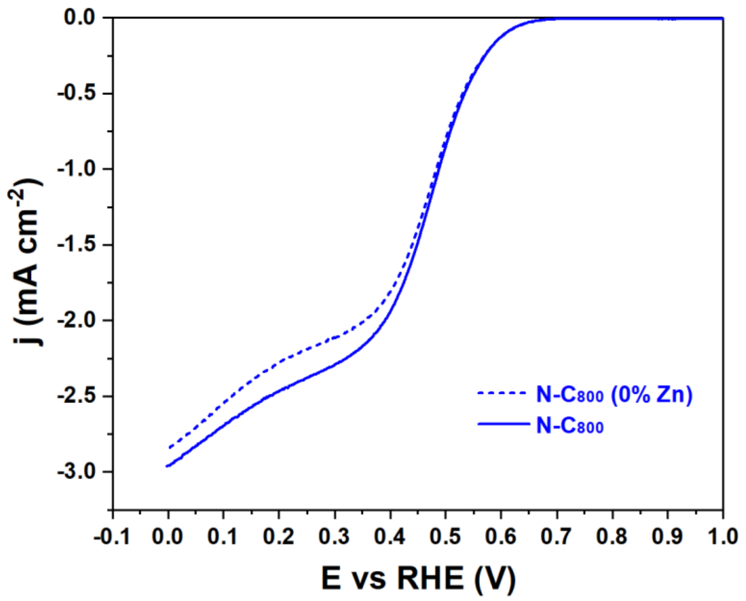

Figure s6 ORR polarization curves of N-C_{800} (0% Zn) and N-C_{800} in O_2-saturated 0.5 M NaCl (rotation rate: 1600 rpm, sweep rate: 10 mV s^{-1}).

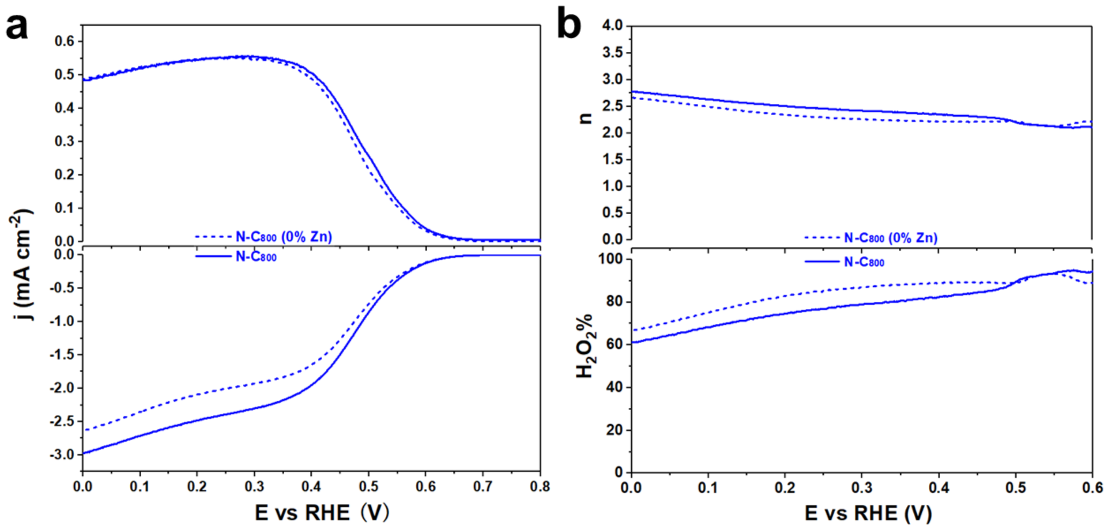

Figure s7 (a) Linear sweep voltammetry (N-C_{800} (0% Zn) and N-C_{800}) performed by a RRDE technique where the ring current is collected on the Pt ring at a constant potential of 1.5 V_{RHE}, (b) the calculated n and H_2O_2 selectivity (%) of N-C_{800} (0% Zn) and N-C_{800}, as a function of electrode potential.

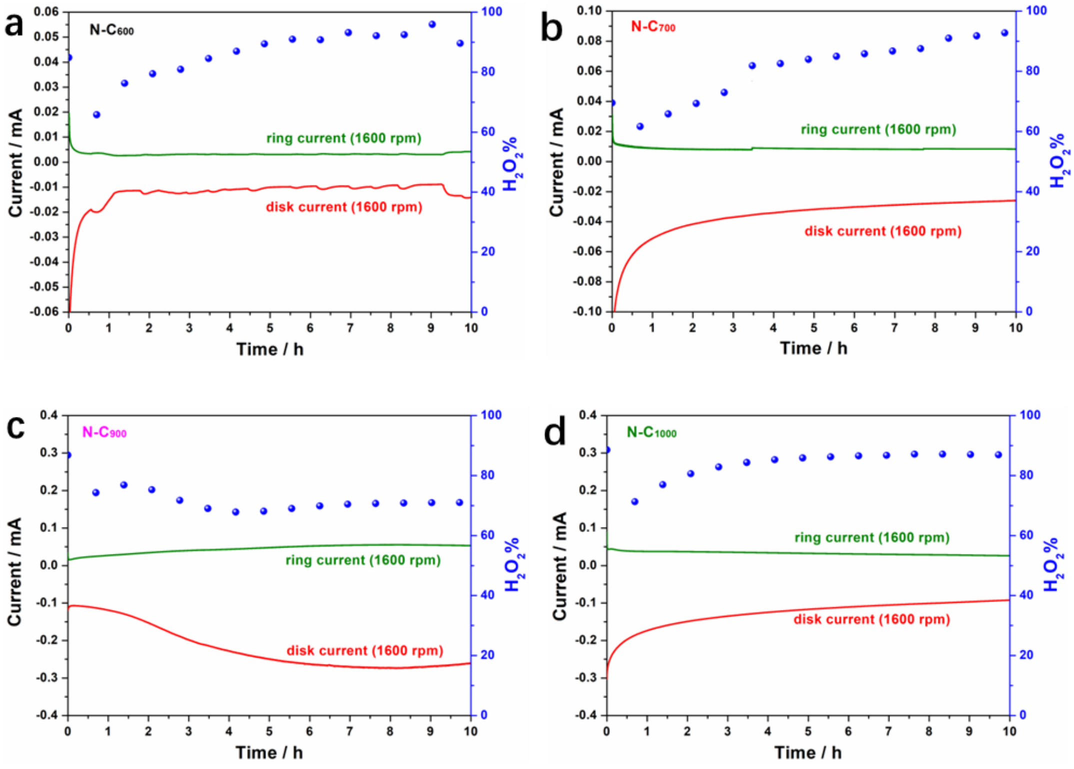

Figure s8 The stability tests of the N-C_x at the different applied potential. (a) N-C_{600}, 0.12 V, (b) N-C_{700}, 0.21 V, (c) N-C_{900}, 0.47 V, (d) N-C_{1000}, 0.17 V.

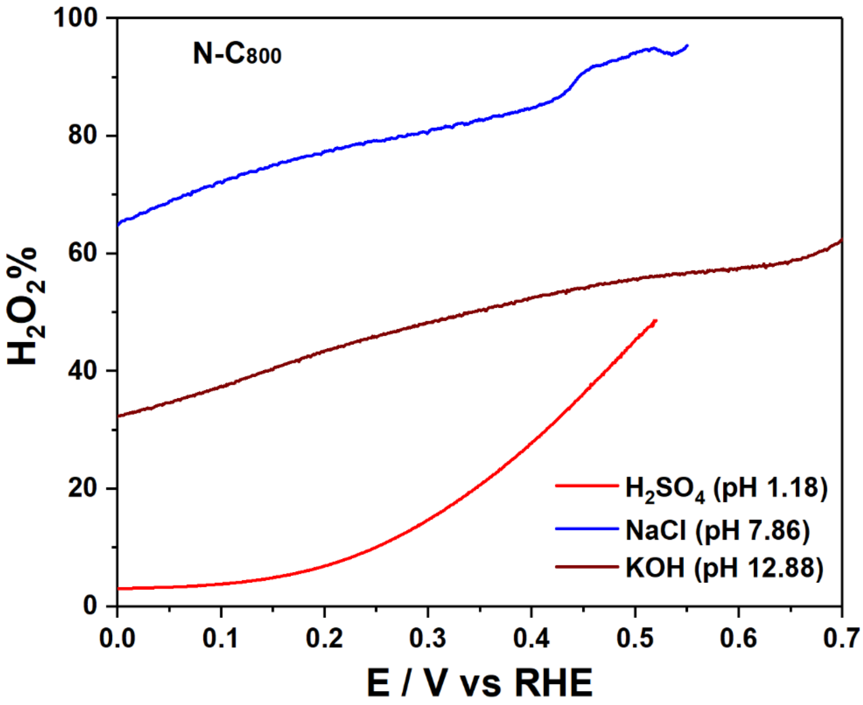

Figure s9 Calculated H_2O_2 selectivity of N-C_{800} in 0.1 M H_2SO_4, 0.5 M NaCl and 0.1 M KOH electrolyte solution, separately.

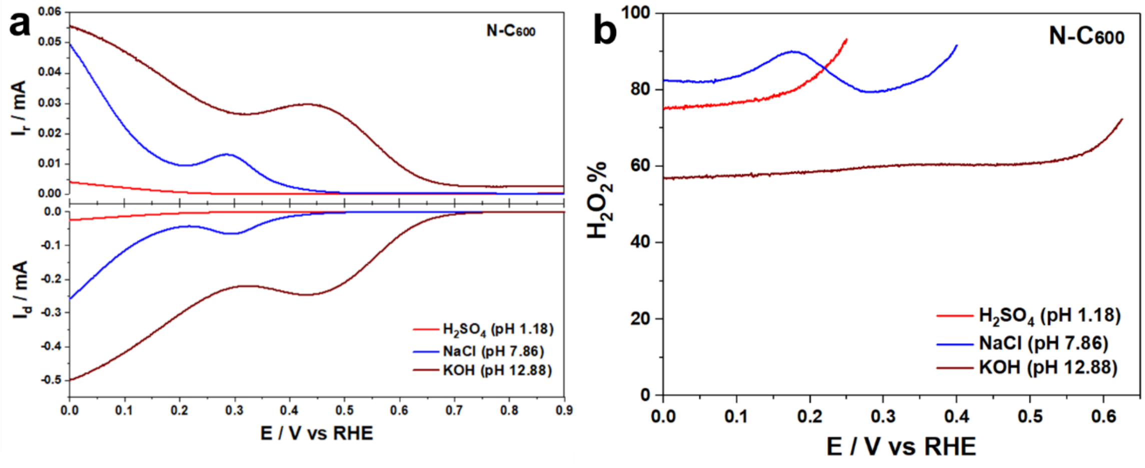

Figure s10 (a) LSV of N-C_{600} recorded in 0.1 M H_2SO_4, 0.5 M NaCl and 0.1 M KOH at 1600 rpm, the ring electrode at a constant potential of 1.5 V_{RHE}, (b) The corresponding calculated H_2O_2 selectivity.

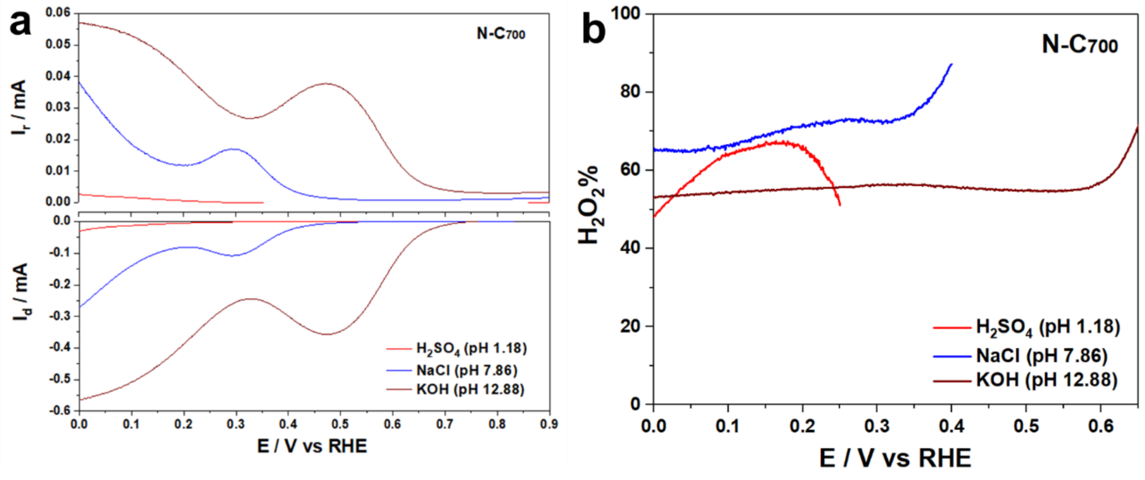

Figure s11 LSV of N-C_{700} recorded in 0.1 M H_2SO_4, 0.5 M NaCl and 0.1 M KOH at 1600 rpm, the ring electrode at a constant potential of 1.5 V_{RHE}, (b) The corresponding calculated H_2O_2 selectivity.

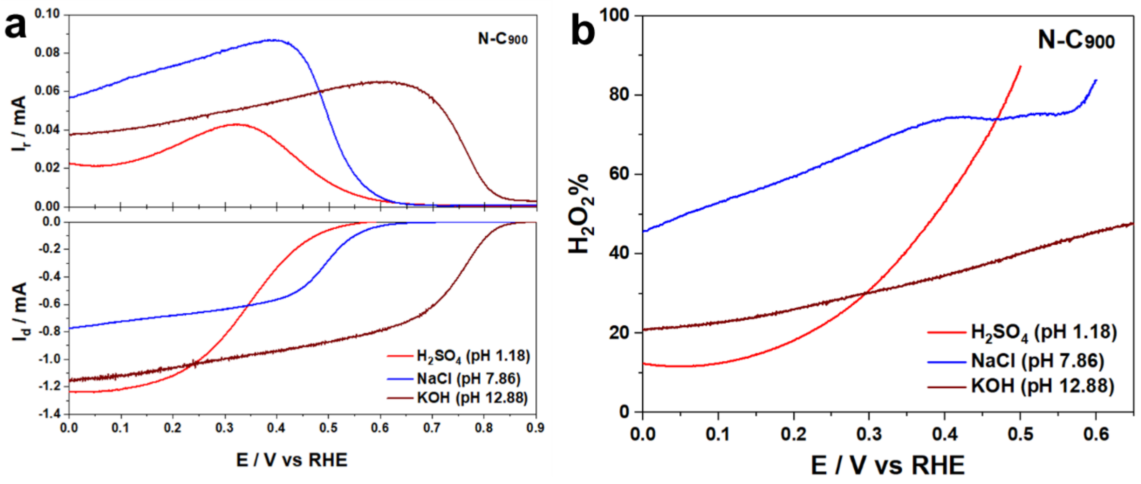

Figure s12 LSV of N-C_{900} recorded in 0.1 M H_2SO_4, 0.5 M NaCl and 0.1 M KOH at 1600 rpm, the ring electrode at a constant potential of 1.5 V_{RHE}, (b) The corresponding calculated H_2O_2 selectivity.

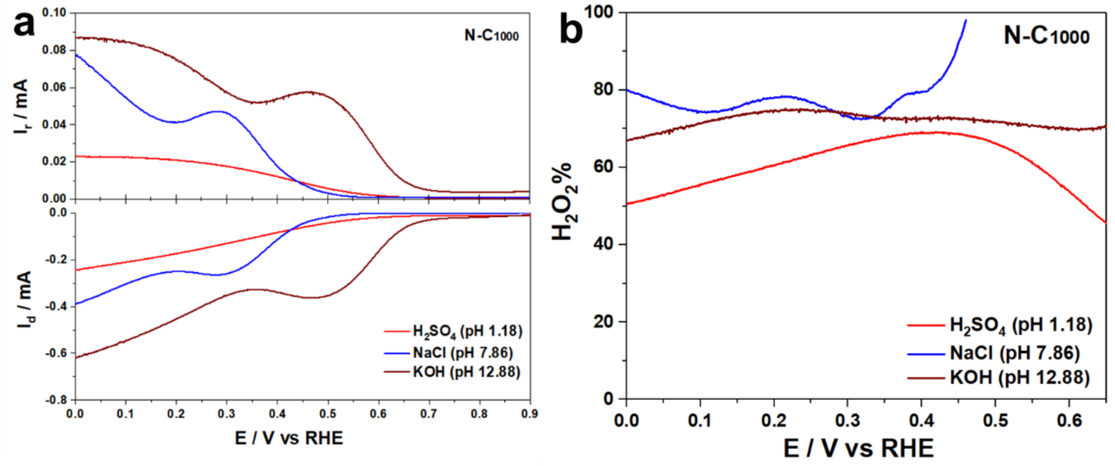

Figure s13 LSV of N-C_{1000} recorded in 0.1 M H_2SO_4, 0.5 M NaCl and 0.1 M KOH at 1600 rpm, the ring electrode at a constant potential of 1.5 V_{RHE}, (b) The corresponding calculated H_2O_2 selectivity.

Table s6 Comparison of this work and recently reported carbon-based catalysts for H_2O_2 production via ORR in neutral electrolytes.

electrocatalysts	Onset potential/ V vs RHE	H_2O_2 yield (%)	Electrolytes	Reference
g-N-CNHs	0.53	95%	0.10 M PBS	[1]
PEI50CMK3-800T	0.52	89.8%	0.1 M K_2SO_4	[2]
O-CNTs	0.53	85%	0.1 M PBS	[3]
NCMK3IL50-800T	0.45	95%	0.1 M K_2SO_4	[4]
CMK3-20s	0.45	78%	0.1M K_2SO_4	[5]
NADE	0.36	88%	0.05 M Na_2SO_4	[6]
Co-N-C	0.6	93%	0.5 M NaCl	[7]
This work	0.601	95%	0.5 M NaCl	This work

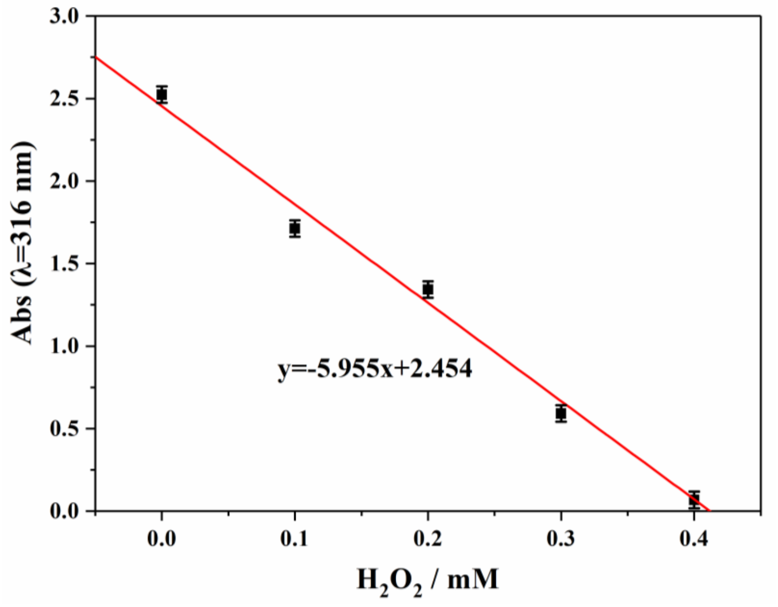

Figure s14 The linear calibration plot for H_2O_2, the error bars shown are the standard errors derived from three measurements.

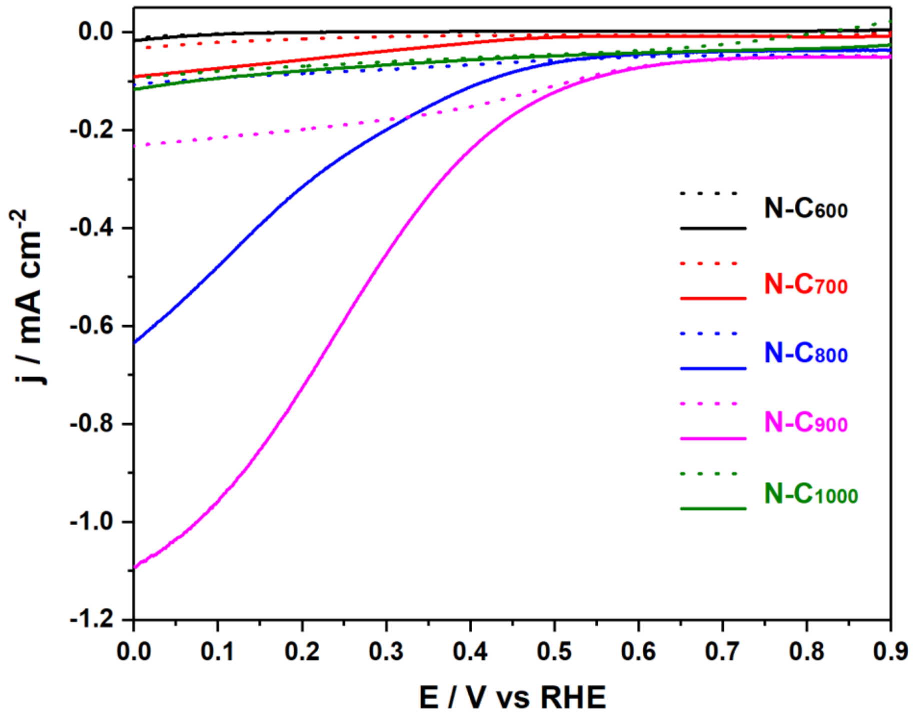

Figure s15 PRR activities of N-C_x were studied by using N_2-saturated 3.5% NaCl electrolyte (dash line) containing 10 mM H_2O_2 (solid line).

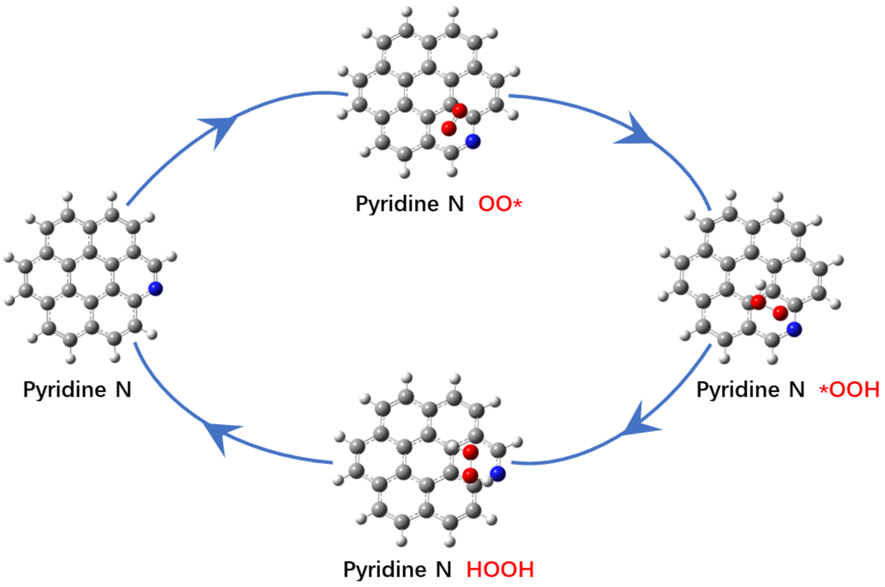

Figure s16 Schematic illustration of the 2e$^-$ pathway on the pyridine N.

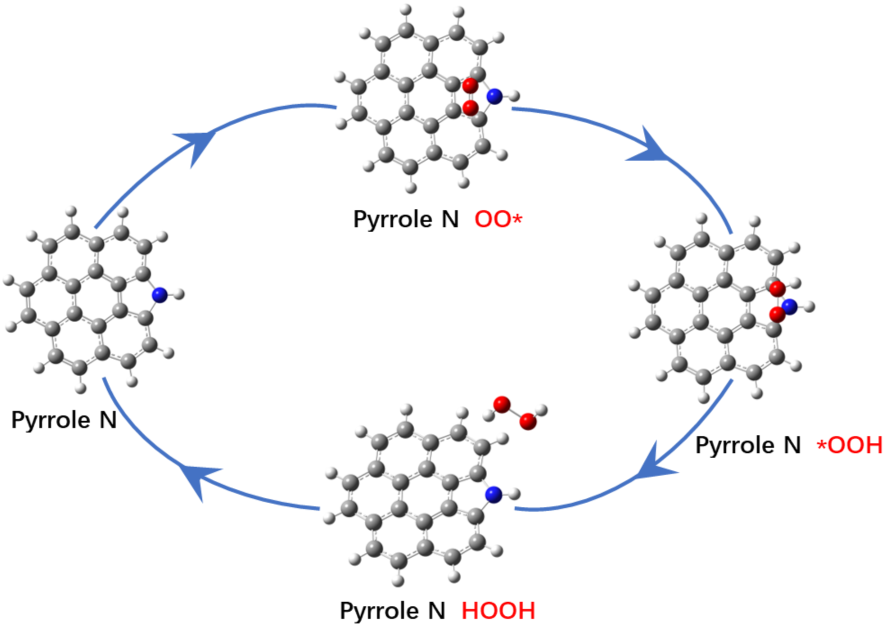

Figure s17 Schematic illustration of the 2e⁻ pathway on the pyrrole N.

Reference

[1] D. Iglesias, A. Giuliani, M. Melchionna, S. Marchesan, A. Criado, L. Nasi, M. Bevilacqua, C. Tavagnacco, F. Vizza, M. Prato, P. Fornasiero, *Chem*, 2018, 4, 106.

[2] Y. Sun, S. Li, Z.P. Jovanov, D. Bernsmeier, H. Wang, B. Paul, X. Wang, S. Kühl, P. Strasser, *ChemSusChem*, 2018, 11, 3388.

[3] Z. Lu, G. Chen, S. Siahrostami, Z. Chen, K. Liu, J. Xie, L. Liao, T. Wu, D. Lin, Y. Liu, T.F. Jaramillo, J.K. Nørskov, Y. Cui, *Nature Catalysis*, 2018,1, 156.

[4] Y. Sun, I. Sinev, W. Ju, A. Bergmann, S. Dresp, S. Kühl, C. Spöri, H. Schmies, H. Wang, D. Bernsmeier, B. Paul, R. Schmack, R. Kraehnert, B. Roldan Cuenya, P. Strasser, *ACS Catalysis*, 2018, 8, 2844.

[5] Y.-L. Wang, S.-S. Li, X.-H. Yang, G.-Y. Xu, Z.-C. Zhu, P. Chen, S.-Q. Li, *Journal of Materials Chemistry A*, 2019, 7, 21329.

[6] Q. Zhang, M. Zhou, G. Ren, Y. Li, Y. Li, X. Du, *Nature Communications*, 2020, 11, 1731.

[7] Q. Zhao, Y. Wang, W.-H. Lai, F. Xiao, Y. Lyu, C. Liao, M. Shao, *Energy & Environmental Science*, 2021, 14, 5444.
